# Test-Retest Reliability of a Food Frequency Questionnaire to Assess Seafood Intake Dynamics for High-End Consumers in Coastal Gulf of Mexico Communities

**DOI:** 10.1155/2020/2704074

**Published:** 2020-04-08

**Authors:** Makyba K. Charles-Ayinde, Leah D. Stuchal, Anne E. Mathews, Andrew S. Kane

**Affiliations:** ^1^Department of Environmental and Global Health, College of Public Health and Health Professions, University of Florida, Gainesville, FL 32611, USA; ^2^Current: American Association for the Advancement of Science, 2415 Eisenhower Avenue, Alexandria, VA 22314, USA; ^3^Center for Environmental and Human Toxicology, University of Florida, 2187 Mowry Rd, Gainesville, FL 32611, USA; ^4^Institute of Food and Agricultural Sciences, Department of Food Science and Human Nutrition, University of Florida, Gainesville, FL 32611, USA; ^5^University of Florida Emerging Pathogens Institute, Gainesville, FL 32611, USA; ^6^Florida Sea Grant Program, Gainesville, FL 32611, USA

## Abstract

**Background:**

Estimates for fish and shellfish intake are used to inform communities and healthcare systems about potential health risks and benefits for individuals, communities, and vulnerable populations. A dietary assessment instrument was designed for use in populations of high-end consumers of seafood to examine intake of finfish, shrimp, oysters, and blue crab in coastal communities across the Gulf of Mexico.

**Objective:**

To validate the reliability of a novel food frequency questionnaire (FFQ) for seafood intake.

**Design:**

Test-retest reliability of the FFQ, which included a species-specific photographic portion guide, was evaluated by the inperson administration and readministration of the instrument with each participant by the same interviewer. Responses from coastal and noncoastal participants were compared to discern FFQ reliability in heterogeneous samples. *Participants/setting*. A convenience sample of 27 coastal participants from Cedar Key, Steinhatchee, and Apalachicola, Florida, reported data for 101 household members; and 15 noncoastal participants from Gainesville, Florida, reported for 42 household members. *Analysis*. Repeated measures from the FFQ were evaluated using correlation concordance for continuous variables (age, weight, and height) and kappa coefficient for categorical variables (type, amount, and frequency of seafood consumed).

**Results:**

Concordance correlation coefficient (1.00) and kappa coefficient (*r* = 0.73 to 1.00) for yearly and seasonal seafood consumption indicated substantial to almost perfect reproducibility, i.e., participants provided responses that were reproducible. Test-retest agreement was highest for coastal participants who consumed more seafood, as compared to occasional, noncoastal consumers, based on the intergroup comparison of kappa coefficients for yearly and seasonal seafood consumption (*r* = 0.69 to 0.99).

**Conclusions:**

The seafood FFQ instrument evaluated in this study, included as a supplement to this report, used in tandem with a photographic portion guide, provides a utilitarian tool for assessing fish, shrimp, oyster, and blue crab intake dynamics in adult and youth populations drawn from coastal communities.

## 1. Introduction

Reliable assessments of seafood consumption are important for monitoring the nutritional status at the community or population level that correlates consumption with potential environmental exposure to contaminated seafood. Measurement of seafood intake therefore is critical in cases when seafood consumption is associated with disease or adverse health outcomes such as cancers, developmental delays, and neurological damage. Common dietary measures to assess the intake of seafood and other foodstuffs include the use of 24-hour recalls, food diaries, biological markers, photo documentation, food frequency questionnaires, as well as a combinatory approach that may provide a more accurate estimate of seafood consumption rates [1, 2].

Food frequency questionnaires (FFQs) have been widely used in epidemiologic and clinical research to assess dietary intake across various populations [3]. FFQs are typically self-reported measures where participants indicate the frequency and amount of their ingestion of commonly consumed foods. FFQs are often chosen over food diaries or multiple 24-hour diet recall studies to estimate consumption rates since data analysis is typically less expensive and time consuming [4]. Efficient and appropriate FFQs would include a logical flow of questions, be less burdensome than daily dietary records, as well as have several food photographs to assist with portion-size estimation [5]. As a result of the decreased burden of FFQs, they have been found to have good psychometric properties including validity and reliability [6]. FFQs may also be preferable to food diaries since food diaries are often backfilled, and participant compliance is generally variable to poor [7]. FFQs tailored for populations with unique dietary patterns have been shown to be important for response accuracy [8]. Although FFQs have many advantages over other forms of data collection, they can lack accurate or precise measures or estimates of food consumption based on recall bias and other factors including clarity of questions for specific audiences. FFQs should be validated with respect to the target group of interest as eating habits and availability of food items, such as seafood, can vary significantly. Consumption patterns can also be dependent on culture, ethnicity, and geography [9]. It is therefore critical that the psychometric properties of the instrument are adequately tested to ensure that estimates provided are realistic measures of the consumption rates of the participants enrolled. In addition, FFQs implemented to assess consumption rates for young children and adolescents require different focus and verbiage than might be used for adult populations. These examples reinforce the need for testing and validation of modified or uniquely developed FFQs for the strength of its psychometric properties.

An instrument validation process includes assessing the reproducibility of measurements from one questionnaire administration to another, i.e., a test-retest examination with the same person(s). Such reliability testing is critical when a new instrument is developed. The ability of the instrument to provide response precision needs to be determined compared with that assessed by the accepted gold standard of measurement. Validity studies typically access the strength of association between an FFQ and another survey type such as a 24-hour recall survey, which is referred to as the criterion measure [4]. Correlation coefficients are calculated to measure the strength of this association.

The overarching Healthy Gulf, Healthy Communities (HGHC) study engaged with the Gulf coast community residents to determine potential human health risks associated with consumption of Gulf-caught seafood following the deepwater horizon oil spill. To assess the association between exposure and risk in this cohort, a specific seafood dietary assessment instrument designed for use in higher-end seafood consumers was required. The seasonality of consumption was also important as seafood consumption patterns can vary based on the availability of certain types of seafood.

Development of this novel FFQ to assess Gulf coastal seafood intake was fashioned after an instrument as described by Toy et al. [10] who designed to capture seafood intake for adolescent and adult high-end consumers in Tulalip and Squaxin Island tribes in the Puget Sound region. The purpose of the current study was to examine the reproducibility of the novel seafood FFQ, for estimating usual seafood intake in representative subsets of adult, adolescent, and child household members who participate, contributing to the HGHC study. The HGHC FFQ was developed in tandem with a previously validated photographic portion guide to support dietary intake for Gulf seafood [11].

## 2. Materials and Methods

### 2.1. Study Population

**Figure 1:**
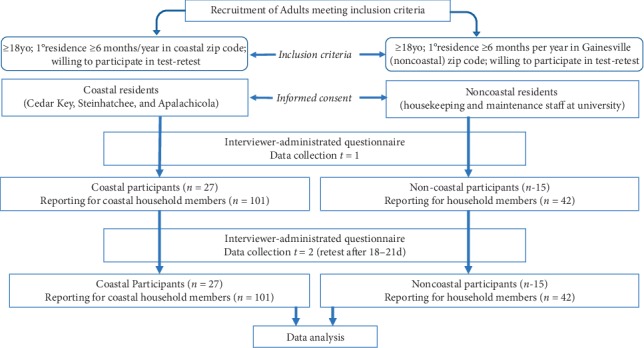
Flow diagram showing validation study design.

Adult participants provided FFQ responses representing themselves and other members in their respective households. Participants represented a convenience sample from three Florida Gulf coastal communities: Cedar Key, Steinhatchee, and Apalachicola and a noncoastal community: Gainesville. Selection of these four regions was not purposive but by convenience. Participants were recruited during March and April 2015 through emails disseminated to physical plant employees of the University of Florida, Gainesville, FL, at the Steinhatchee Florida Seafood festival as well as through outreach facilitated by community partners in Apalachicola and Cedar Key. Recruitment targeted men and women who were 18 years of age or older and who matched the demographic of the target populations residing along the Gulf coast (Apalachicola, Cedar Key, and Steinhatchee areas) or who were university housekeeping and maintenance staff employed by the University of Florida (Figure 1).

### 2.2. Development of the FFQ

The FFQ was designed to measure dietary seafood consumption of children, adolescents, and adults with a reference time for reporting typical seafood consumption with the past three months or “current season,” three months prior to the current season or “last season,” as well as for the “past year,” ending with the time of the FFQ interview. Participants used a validated, photographic seafood portion-size guide [11] to aid in reporting seafood portion sizes to the interviewer. Portion sizes shown in the photographic guide ranged from 2 to 16 ounces in 2-ounce increments. To facilitate data entry, response data for intake amount for each seafood type were field-coded in the survey with relative frequency data fields based on amounts reported: none, <once per month, # times per month, # times per week, or # times per day; data were normalized for analysis to discern intake as grams per day.

### 2.3. Questionnaire Administration

All questionnaires were implemented in-person by the same interviewer who completed training on protocols for administering food frequency questionnaires, in addition to training on the use of the portion size and seafood species identification guides. This study was approved and conducted under the auspices of the University of Florida IRB (protocol no. 333-2011). Informed consent was obtained from all participants before data were collected. The interviewer read each question and corresponding response options aloud, ensuring that there was a sense of comprehension and internalization by the participant. Reimplementation of the in-person questionnaire was separated by an interval of 18–21 days for each participant. A retest interval not exceeding 21 days was considered appropriate as it approximated the typical 14-day interval, typically utilized in test-retest reliability studies [11, 12]. Participants were given a token “thank you gift” of appreciation for the amount of $25 for their time and contribution to the study. Data were recorded in the field using a paper FFQ instrument and were subsequently entered into MS Excel spreadsheets and verified to reduce errors associated with data entry. The FFQ (Figure S1 in the Supplementary Material) is included as a supplement to this report for consideration by other investigators when discerning seafood intake in coastal populations that tend to have relatively higher rates of consumption.

### 2.4. Statistical Analysis

Test-retest reliability between the repeated measures was evaluated using the correlation concordance coefficient for continuous variables (age, weight, and height) and the kappa coefficient for categorical variables (type, amount, and frequency of seafood consumed). Concordance correlation is considered robust with small sample sizes and variable distributions [13]. The descriptive scale for interpretation concordance in this study follows McBride et al. [14]. Fleiss and Cohen weighted Kappa coefficients were used to measure differences between the observed and expected agreement, adjusting for the degree to which responses may agree due to chance [15, 16]. A quadratic weighting scheme was used for its practical interpretation [7] and its equivalence to the intraclass correlation coefficient [8]. Interpretation of the kappa statistic implemented in the study was defined by Landis and Koch [17]. Age data reported for participants were reported as means ± SD. Statistical analyses were performed using the SAS package version 9.3.

### 2.5. Coastal and Noncoastal Intergroup Assessment

A comparison of the performance of the questionnaire for coastal and noncoastal participants was performed to assess the ability of the questionnaire to obtain precise responses irrespective of consumption frequency. Participants from Apalachicola, Cedar Key, and Steinhatchee, Florida, represented 37, 25, and 9% of the Coastal household data, respectively. Participants recruited locally at University of Florida, through campus-wide advertisements targeting housekeeping and maintenance staff, represented the remaining 29% of household data reported from noncoastal household members in this validation study. Inclusion of noncoastal data in this study provided response comparisons between potentially lower- and higher-end consumers of seafood in communities of similar economic means.

## 3. Results

### 3.1. Participant Characteristics

Forty-two participants provided responses for 143 household members from Gulf coastal communities and from Gainesville, FL. Participants reported seafood consumption for 74 male and 69 female household members, with a mean age of 35.6 ± 21.2 years (range = 2–78 years) at the time of data collection. Most household members were Caucasian or African American (Table 1). Mean differences in age and weight reported in repeat FFQ administrations (FFQ_1_–FFQ_2_) were 0.01 years (95% CI −0.09 to 0.1) and −0.8 kg (95% CI −2.8, 1.2), respectively.

### 3.2. Overall Reproducibility

**Figure 2:**
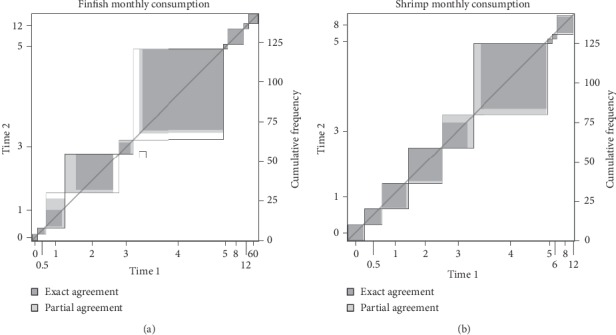
Illustration of the agreement in response to the yearly frequency of the consumption of finfish (a) and shrimp (b) between the two administrations of the questionnaire. Both items displayed an almost perfect agreement based on kappa coefficients of 0.99 and 0.86 (a and b, respectively) in the reliability assessment.

**Figure 3:**
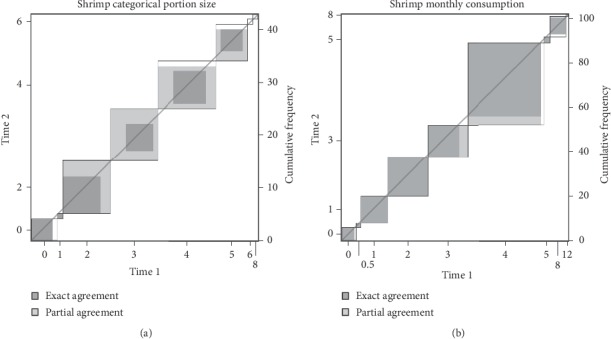
An illustration of the agreement in the responses to the shrimp portion size variable in the noncoastal subgroup (a) and yearly frequency of the consumption of shrimp (monthly) in the coastal subgroup (b). The diagrams represent relatively low level of agreement between the responses.

Age responses for all participants for both FFQ_1_ and FFQ_2_ showed a high degree of correlation with a concordance correlation coefficient of 1.00 (95% CI 0.99, 1.00). Weight responses had substantial correlation with a correlation of 0.98 (95% CI 0.98, 0.99). There is little variation from the concordance line in both age and weight responses when comparing both administrations (FFQ_1_ and FFQ_2_) indicating a high degree of precision. There were no theoretical or extreme consumers in the responses for household members provided by participants which attributed to the high concordance coefficients observed in this study (Figures 2 and 3).

Consumption variables used to test reliability of yearly seafood intake frequencies and typical portion sizes had high weighted kappa coefficients ranging from 0.86 to 1.00 (Table 2), indicating substantial to almost perfect agreement for all questions that would be used to derive yearly seafood consumption rates for finfish, shrimp, oyster, and blue crab. The consumption variable “*how often you ate fish over the entire past year?*” reported in monthly and daily frequencies, had the highest reliability (1.00), with a marked amount of exact agreement in responses from FFQ_1_ compared to FFQ_2_ (dark regions). The item “*how often you ate shrimp over the entire past year?*” reported in monthly frequencies had the lowest reliability (0.86). Although there appears to be a marked amount of exact agreement in responses from FFQ_1_ compared to FFQ_2_ (dark regions), the presence of partial agreements which are further apart carried less weight resulting in a low-weighted kappa coefficient.

Variables to discern seasonal consumption rates of finfish, shrimp, oyster, and blue crab during the “current season” and the “last season” had weighted kappa coefficients ranging from 0.73 to 1.0 (Table 3), indicating substantial to almost perfect agreement for all consumption variables associated with the derivation of seasonal seafood consumption rates. The item “*how often you ate fish during the current season (last three months)*?” reported in daily frequency had the highest reliability (1.00). The item “*how often you ate shrimp during the current season (last three months)*?” reported in daily consumption frequency had the lowest reliability (0.73).

### 3.3. FFQ Reliability Assessment for Coastal versus Noncoastal Participants

Intergroup FFQ reliability was compared between coastal and noncoastal study participants. There were 27 participants from coastal communities who provided responses for 101 household members (49 males and 52 females); mean age for coastal household members was 33.7 ± 22.1 yo (range = 2–78 yrs) at the time of data collection; 32 were African American, and 69 were Caucasian. There were 15 participants from noncoastal communities who provided responses for 42 household members (25 males and 17 females); the mean age for coastal household members was 40.3 ± 18.1 at the time of data collection; 18 were African American, 4 were Hispanic, 14 were Caucasian, and 6 were Asian (Table 1). Age responses for were highly correlated in both coastal and noncoastal subgroups with concordance correlation coefficients of 0.99 (95% CI 0.99, 1.00) and 0.99 (95% CI 0.99, 1.00), respectively. Weight responses were also highly correlated in both groups with concordance correlation coefficients of 0.99 (95% CI 0.98, 0.99) and 0.97 (95% CI 0.94, 0.98), respectively. There was little variation from the concordance line in both age and weight illustrating a great degree of precision.

Kappa coefficients for yearly seafood consumption frequency and typical portion sizes ranged from 0.80 to 0.99 and 0.63 to 1.00, for coastal and noncoastal participants, respectively, indicating substantial to almost perfect agreement between responses for participants from the noncoastal household members (Table 4). Yearly seafood consumption responses from coastal and noncoastal household members had mean weighted kappa coefficients of 0.93 and 0.90, respectively. Shrimp intake data provided the lowest kappa values observed in this study: 0.80 and 0.63 for coastal and noncoastal participants, respectively (Table 5).

Seasonal consumption of fish, shrimp, oyster, and blue crab had weighted kappa coefficients ranging from 0.69 to 0.99 and 0.65 to 1.00 for coastal and noncoastal participants, respectively (Table 6). This indicates substantial to almost perfect agreement for all seasonal consumption variables for both coastal and noncoastal household members in the study. Actual dietary intake for both coastal and noncoastal participants in this instrument validation study, for all seafood types, indicated almost identical test-retest results (Table 7).

## 4. Discussion

This is the first study to report test-retest reliability of an FFQ designed for implementation in Gulf coast communities where eating habits, seafood availability, and seafood as part of the culture are important. To estimate consumption rates, participants would need to accurately recall both frequencies and amounts of seafood consumption. Under- or overestimation or the inability to consistently recall their consumption patterns may occur due to the availability of seafood resources and the variety of sources through which they obtain seafood for consumption. Therefore, the reliability of the instrument should be tested.

Results of the test-retest reliability showed excellent reproducibility between repeated administrations of the FFQ, suggesting the questionnaire is a reliable method of assessing habitual seafood consumption in Gulf coast communities, i.e., the instrument was able to derive precise recall data from participants. Reliability on consumption estimates ranged from a weighted kappa coefficient of 0.73 to 1.00, indicating that there is substantial to almost perfect agreement between responses, indicative of a reliable food frequency instrument. Age and weight responses had the highest level of reliability (mean correlation coefficient of 0.99).

Amongst all participants, yearly consumption rate items were slightly more reliable than seasonal consumption rate items with mean weighted kappa coefficient of 0.94 and 0.93, respectively. In other test-retest studies of FFQs examining consumption of fish and shellfish, kappa coefficients range from 0.45 to 0.75 [18–20]. This FFQ showed relatively higher kappa coefficients, indicating good reproducibility and reliability. The length of the interval between the two administrations may have contributed to the high reliability observed. Studies with an interval between repeated FFQ administrations less than one month gave higher reliability coefficients than those further apart [21]. Other test-retest studies using a 3-month interval between the two administrations generally had lower kappa values for fish and shellfish consumption (0.45–0.62) suggesting moderate to substantial agreement [18, 19]. The reproducibility study on the dietary habits of Polish adolescents and adults used a test-retest interval of 14 days, which is similar to the current study test-retest interval of 21 days. The Polish study reported a kappa statistic of 0.75 for fish consumption on the interviewer-administered questionnaire [20]. Careful consideration was also made to ensure that the seasonal section of the questionnaire assessed the same time period for both administrations. This also ensured that any influences of seasonal food availability and dietary changes were minimized.

The ability of this FFQ to capture highly reliable responses (substantial to almost perfect agreement) for yearly consumption rates makes it a useful tool for assessing habitual seafood consumption in coastal communities. Traditionally, studies with shorter time span assessment periods (i.e., assessment of food intake from the previous day or week) had better reliability compared to those with longer periods, such as the yearly consumption rates, as assessed by this questionnaire [4]. The tailoring of the questionnaire to the target population (coastal participants) may have attributed to the ability of the instrument to reliably capture yearly seafood consumption rates, but traditionally found with these FFQ types. Consideration of a culturally relevant food choice as well as cognitive design and administration specific to this population would have also contributed to the reliability of the instrument. The development of the FFQ to be of a medium length and be completed within a 15-minute period may have also attributed to the high reliability observed. A previous study demonstrated that there is an association between the number of questionnaire items and the strength of correlation and reliability. Studies with a medium length questionnaire, up to 63 items, had the strongest reliabilities [4]. The length of the HGHC questionnaire, 48 items, is therefore long enough to assess an adequate amount of information; however, it is short enough to not result in participant burden and subsequent reporting error.

In general, items which assessed portion sizes had low-weighted kappa values. This is consistent with patterns observed in previous reliability studies. Studies which did not assess portion size had higher correlations with the reference criterion and reliability than those that did or those that partially assessed portion sizes. Reliability testing that did assess portion size, however, showed correlations that ranged from weak to moderate [4]. This is also observed in the noncoastal subgroup, where the shrimp portion size item had a weighted kappa coefficient of 0.6347, one of the lowest reliabilities observed throughout both seafood consumption sections (yearly and seasonal).

Test-retest reliability of the FFQ between the coastal and noncoastal subgroups was used to compare the generalizability for using the instrument. The food frequency questionnaire was more reliable for coastal participants compared to the noncoastal participants in all items tested. Typically, such differences might be associated with age since older adults tend to have more established and stable diets compared with younger adults, thus reducing variation in intraindividual eating habits [21]. This was not the case for the coastal and noncoastal household members in the present study with mean ages of years 33.7 and 40.3 years, respectively. Perhaps the cultural and economic importance, and generally higher intake rates, of seafood consumption for coastal participants, compared to noncoastal participants, supported for more precise recall data. This is based on fewer commercial and recreational fishers within the noncoastal subgroup surveyed, who more likely access seafood through retail purchases rather than having direct access to fresh Gulf seafood. As a result, fluctuations in consumption may not be based on seasonality but instead driven more by economic and cultural differences within this group. Economic fluctuations and its impact on consumption may be more difficult to recall compared to changes in availability of seafood. This would have directly impacted the reliability of the questionnaire and is reflected in the more modest test-retest agreement in the noncoastal subgroup.

### 4.1. Limitations

Results from this pilot study could have been improved by collecting and analyzing other demographic variables, including participant or household member income levels, and increasing the number of participants, particularly in light of the wide age range of household members reported. While overall sample size may have been limited in this validation study (*n* = 42), this sample size is comparable with similar validation studies in the literature [4, 5, 9, 12, 22], and power analyses for monthly and daily intake responses were 0.999 for all seafood types. Categorical variables (portion sizes), however, had lower power 0.889, 0.986, 0.993, and 0.679 for fish, shrimp, oyster, and blue crab, respectively (Table 6), suggesting a greater sample size may have improved outcomes from this study.

The FFQ was administered without the support of a food diary or weighed food records, and it was therefore not possible to validate response accuracy of the FFQ as there was no measure of “true” seafood consumption rates. Intermethod reliability, which is a comparison between a repeated dietary recall and the FFQ, would be an appropriate comparison to determine the validity of this FFQ to capture accurate and precise seafood consumption rates. At this time, only the reliability of the instrument along with a previously validated photographic portion guide [11] was assessed to determine the effectiveness of the instrument to estimate the precision of individual household member seafood consumption rates.

## 5. Conclusion

We demonstrated reproducibility of a novel and utilitarian food frequency questionnaire that includes a photographic seafood portion guide, designed for assessing seafood intake and consumption patterns for youth and adult household members in coastal Gulf of Mexico communities relative to seafood safety. This highly tailored dietary assessment tool was effective in estimating seafood intake (based on high concordance correlation and kappa statistics). The FFQ provided similar precision across ethnically heterogeneous populations with diverse seafood consumption patterns, making it a utilitarian instrument for exposure assessment. Due to its high reliability in both coastal and noncoastal subgroups, across a wide range of portion sizes, the FFQ reported in this study could be generalizable to other populations with similar characteristics as our targeted Gulf coast community populations.

## Figures and Tables

**Table 1:** Demographics (numbers and percentages) for coastal and noncoastal household members reported by study participants and mean age (±SD) for participants who contributed data for themselves and their households.

	Coastal household data (*n* = 101, 71%)		Noncoastal household data (*n* = 42, 29%)	
	*Reported from 27 Participants* (%)		*Reported from 15 Participants* (%)	
Gender				
Female	52	49	17	41
Male	49	51	25	59

Age				
<15	30	30	6	14
15–24	11	11	3	7
25–44	20	20	13	31
45–65	32	32	18	42
>65	8	8	2	5

Race/ethnicity				
African American	32	32	18	43
Asian	0	0	6	14
Caucasian	60	68	14	33
Hispanic	0	0	4	10

Age of contributing participants	41.7 ± 17.8		42.9 ± 16.8	

**Table 2:** Weighted kappa coefficients obtained in the reliability analyses on survey questions that discerned *yearly* fish, shrimp, oyster, and blue crab consumption frequencies, as well as respective portion sizes. Weighted kappa coefficients for all test-retest outputs had *p* < 0.0001.

	Consumption variable	Weighted kappa coefficient	95% CI
Fish	Yearly consumption (month)	1.00	0.99‐1.00
	Yearly consumption (day)	1.00	0.99‐1.00
	Portion size	0.92	0.89–0.96

Shrimp	Yearly consumption (month)	0.86	0.67–1.00
	Yearly consumption (day)	0.86	0.67–1.00
	Portion size	0.94	0.91–0.97

Oyster	Yearly consumption (month)	0.97	0.95–0.99
	Yearly consumption (day)	0.97	0.95–0.99
	Oyster portion size	0.95	0.91–0.98

Blue crab	Yearly consumption (month)	0.96	0.94–0.99
	Yearly consumption (day)	0.96	0.93–0.99
	Portion size	0.87	0.79–0.94

**Table 3:** Weighted kappa coefficients for household members reported by all study participants for *seasonal* finfish, shrimp, oyster, and blue crab consumption rates. Weighted kappa coefficient for all test-retest outputs was <0.0001.

	Consumption variable	Weighted kappa coefficient	95% CI
Fish	Current season (month)	1.00	0.99‐1.00
	Current season (day)	1.00	0.99‐1.00
	Last season (month)	1.00	0.99‐1.00
	Last season (day)	1.00	0.99‐1.00

Shrimp	Current season (month)	0.74	0.51–0.98
	Current season (day)	0.73	0.49–0.97
	Last season (month)	0.83	0.65–1.00
	Last season (day)	0.82	0.65–1.00

Oyster	Current season (month)	0.97	0.94–1.00
	Current season (day)	0.97	0.93–1.00
	Last season (month)	0.97	0.94–1.00
	Last season (day)	0.97	0.93–1.00

Blue crab	Current season (month)	0.95	0.89–1.00
	Current season (day)	0.94	0.88–1.00
	Last season (month)	0.97	0.95–0.99
	Last season (day)	0.97	0.94–0.99

**Table 4:** Weighted kappa coefficients for coastal and noncoastal household members' typical *yearly*, daily, and monthly consumption estimates and typical portion sizes of finfish, shrimp, oyster, and blue crab. Weighted kappa coefficients for all test-retest outputs had *p* < 0.0001.

	Consumption variable	Coastal	Noncoastal
		Weighted kappa coefficient	Weighted kappa coefficient
Fish	Yearly consumption (month)	0.95	1.00
	Yearly consumption (day)	0.95	1.00
	Portion size	0.96	0.89

Shrimp	Yearly consumption (month)	0.80	0.98
	Yearly consumption (day)	0.81	0.98
	Portion size	0.96	0.63

Oyster	Yearly consumption (month)	0.98	0.89
	Yearly consumption (day)	0.98	0.86
	Oyster portion size	0.94	0.91

Blue crab	Yearly consumption (month)	0.99	0.83
	Yearly consumption (day)	0.98	0.85
	Portion size	0.87	0.97

**Table 5:** Weighted kappa coefficients for coastal and noncoastal household members' typical *yearly*, daily, and monthly consumption estimates and typical portion sizes of finfish, shrimp, oyster, and blue crab. Weighted kappa coefficients for all test-retest outputs had *p* < 0.0001. Power calculations for sample size of *n* = 42, based on results on Pearson's correlation from Fisher's *z*-test for monthly, daily, and portion categories are included for each seafood type.

dsds	Consumption Variable	Coastal	Noncoastal	Sample power (based on *n* = 42)
		Weighted kappa coefficient	Weighted kappa coefficient	
Fish	Yearly consumption (month)	0.95	1.00	>0.999
	Yearly consumption (day)	0.95	1.00	>0.999
	Portion size	0.96	0.89	>0.889

Shrimp	Yearly consumption (month)	0.80	0.98	>0.999
	Yearly consumption (day)	0.81	0.98	>0.999
	Portion size	0.96	0.63	>0.986

Oyster	Yearly consumption (month)	0.98	0.89	>0.999
	Yearly consumption (day)	0.98	0.86	>0.999
	Oyster portion size	0.94	0.91	>0.993

Blue crab	Yearly consumption (month)	0.99	0.83	>0.999
	Yearly consumption (day)	0.98	0.85	>0.999
	Portion size	0.87	0.97	>0.697

**Table 6:** Weighted kappa coefficients for coastal and noncoastal household members' *seasonal* consumption rates for finfish, shrimp, oyster, and blue crab. Weighted kappa coefficients for all test-retest outputs had *p* < 0.0001.

	Consumption variable	Coastal	Noncoastal
		Weighted kappa coefficient	Weighted kappa coefficient
Fish	Current season (month)	0.98	1.00
	Current season (day)	0.98	1.00
	Last season (month)	0.98	1.00
	Last season (day)	0.99	1.00

Shrimp	Current season (month)	0.69	0.69
	Current season (day)	0.69	0.67
	Last season (month)	0.93	0.69
	Last season (day)	0.93	0.67

Oyster	Current season (month)	0.97	0.72
	Current season (day)	0.98	0.65
	Last season (month)	0.97	0.74
	Last season (day)	0.97	0.75

Blue crab	Current season (month)	0.96	0.81
	Current season (day)	0.96	0.79
	Last season (month)	0.99	0.81
	Last season (day)	0.99	0.81

**Table 7:** Mean values (converted to grams) and interquartile ranges (Q25–Q75) for test and retest dietary intake data for finfish, shrimp, oyster, and blue crab, from coastal and noncoastal household members. Differences between initial test data and follow-up retest data for each seafood type, in both participant groups, were not detected with *α* = 0.05 using Mann–Whitney *U*-test (*p* values ranged from 0.61–0.93).

	Coastal participants				Noncoastal participants			
	*Test*		*Retest*		*Test*		*Retest*	
*Seafood type*	Mean	Q25-Q75	Mean	Q25-Q75	Mean	Q25-Q75	Mean	Q25-Q75
Fish	3.4	2.0–3.7	3.4	2.0–3.7	10.5	2.0–3.7	10.2	0.9–3.7
Shrimp	3.1	2.0–3.7	3.1	2.0–3.7	2.6	0.6–3.7	2.6	0.6–3.7
Oyster	1.1	0.0–2.0	1.1	0.0–0.9	0.6	0.0–0.6	0.6	0.0–0.6
Blue crab	0.9	0.0–0.9	0.9	0.0–0.9	0.6	0.0–0.9	0.6	0.0–0.6

## Data Availability

Survey response data used to support the findings of this study have not been made available to protect participant privacy. Participant's informed consent, under the auspices of the University of Florida Institutional Review Board, indicated reporting of data on aggregate to protect the identities of individuals, particularly those living in small communities. Other data used to support the findings of this study are available from the corresponding author upon request.

## Supplementary Materials

Supplementary Materials Food frequency questionnaire and data sheets for fish, shrimp, oyster, and blue crab intake evaluated in the present test-retest reliability study (Figure 1S).
