# Supplementary material for: Test-Retest Reliability of a Food Frequency Questionnaire to Assess Seafood Intake Dynamics for High-End Consumers in Coastal Gulf of Mexico Communities
Source: J Environ Public Health. 2020 Apr 8;2020:2704074. doi: 10.1155/2020/2704074 (PMC7168735; doi:10.1155/2020/2704074)
Supplement: Supplementary Materials — Food frequency questionnaire and data sheets for fish, shrimp, oyster, and blue crab intake evaluated in the present test-retest reliability study (Figure 1S). [file 2704074.f1.pdf]

Figure 1S. Food frequency questionnaire and data sheets for fish, shrimp, oyster and blue crab intake evaluated in the present test-retest reliability study.

SEAFOOD CONSUMPTION SURVEY

Location represented by participant: ______________________________________
Interviewer initials: ____________________________ Survey #: ______________
Date: ____________________________________ Page #: *Page 1 of 9*

Instrument implementation only after participant meets inclusion criteria, and reviews and agree and signing ICF***

Hello, my name is _________ with ______Program from ________. I am part of a research team that is conducting a survey to better understand what seafood people eat from the Gulf. In particular, we are looking at fish, shrimp, crab, and oysters that come from Gulf waters. May I ask you a few questions about the seafood that you catch and eat from the Gulf?

Do you or anyone in your household eat any seafood that you **know was caught** from the Gulf? This includes any fish, shrimp, oyster and crabs that came from the Gulf, and eaten as appetizers, portions of meals, or snacks.

☐ Yes

☐ No

If yes, → Informed consent; continue with survey.
If no, → Thank you very much. Good luck fishing/Have a good day.

What is the zip code of your primary residence (where you live ≥6 months out of the year)? (________________)

☐ DON'T KNOW / REFUSED

How would you best describe yourself with respect to race or ethnicity?

**You may check (✓) more than one box.**	
❑	**Black or African American.** A person having origins in any of the Black racial groups of Africa.
❑	**Hispanic or Latino.** A person of Cuban, Mexican, Puerto Rican, South or Central American, or other Spanish culture or origin, regardless of race.
❑	**White.** A person having origins in any of the original peoples of Europe, the Middle East, or North Africa.
❑	**Native American or American Indian or Alaska native.** A person having origins in any of the original peoples of North, Central and South America, and who maintains tribal affiliation or community attachment.
❑	**Asian.** A person having origins in any of the original peoples of the Far East, Southeast Asia, or the Indian subcontinent including for example, Cambodia, China, India, Japan, Korea, Malaysia, Pakistan, the Philippine Islands, Thailand and Vietnam.
❑	**Native Hawaiian or other Pacific Islander.** A person having origins in any of the original peoples of Hawaii, Guam, Samoa, or other Pacific Islands.
❑	**Other.** A group not mentioned above.
**If *Other* is checked, please describe:**	

First, how many people are there in your household, including yourself and all children? A household includes you any people who regularly live with you, regardless of their relationship to you, so that could include a roommate or friend.

Adults: ____ (Number) Children:_____(Number)

Starting with the adults, could you please tell me their ages, male/female, and body weights, estimating as closely as you can?

Now, I would like to ask you about the specific types of seafood from the Gulf that you and your household members typically eat.

I have some pictures to help you identify the types of fish you eat from the GULF. I'll also ask you about shrimp, crabs, oysters and clams.

First, let's check which types of fish and seafood you ate in the last seven days and in the last year. Next, please identify the five types of seafood you ate most frequently in the last year. For instance, if you ate more shrimp than anything else, then put 1 by shrimp; if you next you ate mackerel, then put a 2 by mackerel and so on.

(Show list of seafood with check list for last week and last year and allow participant to check off all that he/she consumed. Use **Photographic Guide** to help identify the correct coastal fish species, as needed.

FISH (If indicated. If not, indicated, skip to next seafood type.): You indicated that you ate fish from the GULF last week. How often did you eat fish from the Gulf last week? Remember, we are only interested in fish that YOU caught or you KNOW came from the Gulf. Just to be clear, a meal consists of ANY serving, regardless of it being the main part of the meal, or part of a side dish, soup or appetizer - we need to account for all seafood that you eat. **(NOTE**: if a seafood type such as fish, shrimp, crab, oysters, clams is not indicated on the check list, skip that frequency question.)

For the following questions, record participant data on the Data Collection Form

In the past week, how often on average did you eat fish that was caught in the Gulf?

When you eat fish from the Gulf, how much did you typically eat in the past week? Please use these pictures to help you best estimate the amount you usually eat.

Now, please think about how often you ate **FISH** FROM THE GULF during the past year. If you ate more fish some months and less fish other months, please try to estimate how much you ate over the 12 months.

In the past year, how often on average did you eat fish that was caught in the Gulf?

Thank you. When you eat fish from the Gulf, how much did you typically eat? Please use these pictures to help you best estimate the amount you usually eat.

Now I would like to ask you to estimate the amount of **GULF fish** that other members of your household eat. Let's start with the other adults in your household. Please think about how often ____ (adult 1) ate fish FROM THE GULF during the past year. If he/she ate more fish some months and less fish other months, please try to estimate how much he/she ate over the past 12 months.

In the past year, how often on average did he/she eat **Fish** that was caught in the Gulf?

Thank you. When he/she ate fish from the Gulf, how much did he/she typically eat? Please use these pictures to help you best estimate the amount he/she usually ate.
(If there are additional adults, repeat the above questions about fish.)

You indicated there are ___ children in your household. Please think about how often ____ (child 1) ate fish FROM THE GULF during the past year. If he/she ate more fish some months and less fish other months, please try to estimate how much he/she ate – *on average* – over the past year.

In the past year, how often on average did he/she eat fish that was caught in the Gulf?

Thank you. When he/she ate fish from the Gulf, how much did he/she typically eat? Please use these pictures to help you best estimate the amount he/she usually ate.

Thank you. Now, let's do the same for shrimp.

<u>SHRIMP</u> (If indicated. If not, indicated, skip to next seafood type.):
You indicated that you ate shrimp from the GULF last week. How often did you eat shrimp from the Gulf last week? Remember, we are only interested in shrimp that YOU caught or you KNOW came from the Gulf. Just to be clear, a meal consists of ANY serving, regardless of it being the main part of the meal, or part of a side dish, soup or appetizer - we need to account for all seafood that you eat.

For the following questions, record participant data on the Data Collection Form

In the past week, how many times on average did you eat shrimp that was caught in the Gulf?

When you eat shrimp from the Gulf, how much did you typically eat **in the last 7 days**? Please use these pictures to help you best estimate the amount you usually eat.

Now, please think about how often you ate shrimp FROM THE GULF during the past <u>year</u>. If you ate more shrimp some months and less shrimp other months, please try to estimate how much you ate over the 12 months.

In the past year, how often on average did you eat shrimp that was caught in the Gulf?

Thank you. When you eat **shrimp** from the Gulf, how much do you usually eat? Please use these pictures to help you best estimate the amount you typically eat.

Now I would like to ask you to estimate the amount of GULF shrimp that other members of your household eat.

Let's start with the other adults in your household. Please think about how often ____ (adult 1) ate shrimp FROM THE GULF during the past <u>year</u>. If he/she ate more shrimp some months and less shrimp other months, please try to estimate how much he/she ate – *on average* - over the past year.

In the past year, how often on average did he/she eat shrimp that was caught in the Gulf? **(Data Collection Form)**

Thank you. When he/she ate shrimp from the Gulf, how much did he/she typically eat? Please use these pictures to help you best estimate the amount he/she usually ate.

(If there are additional adults, repeat the above questions about shrimp.)

You indicated there are ___ children in your household. Please think about how often ____ (child 1) ate **Shrimp** FROM THE GULF during the past <u>year</u>. If he/she ate more shrimp some months and less shrimp other months, please try to estimate how much he/she ate over the past 12 months.

In the past year, how often on average did he/she eat shrimp that was caught in the Gulf?

Thank you. When he/she ate shrimp from the Gulf, how much did he/she typically eat? Please use these pictures to help you best estimate the amount he/she usually ate.

__**BLUE CRAB**__ (If indicated. If not, indicated, skip to next seafood type.): You indicated that you ate crab from the GULF last week. How often did you eat crab from the Gulf last week? Remember, we are only interested in crab that YOU caught or you KNOW came from the Gulf. Just to be clear, a meal consists of ANY serving, regardless of it being the main part of the meal, or part of a side dish, soup or appetizer- we need to account for all seafood that you eat.

For the following questions, record participant data on the Data Collection Form

In the past week, how often on average did you eat crab that was caught in the Gulf?

When you eat crab from the Gulf, how much did you typically eat **in the last 7 days**? Please use these pictures to help you best estimate the amount you usually eat.

Now, please think about how often you ate crab FROM THE GULF during the past __year__. If you ate more crab some months and less crab other months, please try to estimate how much you ate over the 12 months.

In the past year, how often on average did you eat crab that was caught in the Gulf?

Thank you. When you eat crab from the Gulf, how much did you usually eat? Please use these pictures to help you best estimate the amount you typically eat.

Now I would like to ask you to estimate the amount of GULF crab that other members of your household eat.

Let's start with the other adults in your household. Please think about how often ____ (adult 1) ate crab FROM THE GULF during the past __year__. If he/she ate more crab some months and less crab other months, please try to estimate how much he/she ate over the past 12 months.

In the past year, how often on average did he/she eat crab that was caught in the Gulf?

Thank you. When he/she ate crab from the Gulf, how much did he/she typically eat? Please use these pictures to help you best estimate the amount he/she usually ate.

(If there are additional adults, repeat the above questions about crab.)

You indicated there are ___ children in your household. Please think about how often ____ (child 1) ate crab FROM THE GULF during the past __year__. If he/she ate more crab some months and less crab other months, please try to estimate how much he/she ate over the past 12 months.

In the past year, how often on average did he/she eat **Crab** that was caught from the Gulf?

Thank you. When he/she ate fish from the Gulf, how much did he/she typically eat? Please use these pictures to help you best estimate the amount he/she usually ate.

<u>OYSTERS</u> (If indicated. If not, indicated, skip to next seafood type.):
You indicated that you ate oysters from the GULF last week. How often did you eat oysters from the Gulf last week? Remember, we are only interested in oysters that YOU caught or you KNOW came from the Gulf. Just to be clear, a meal consists of ANY serving, regardless of it being the main part of the meal, or part of a side dish, soup or appetizer- we need to account for all seafood that you eat.

For the following questions, record participant data on the Data Collection Form

In the past week, how many times on average did you eat oysters that were caught in the Gulf?

When you ate oysters from the Gulf last week, how much did you eat? Please use these pictures to help you best estimate the amount you ate last week. If you ate oysters more than once last week, please select your average portion size when considering each time you ate oysters last week.

Now, please think about how often you ate oysters FROM THE GULF during the past <u>year</u>. If you ate more oysters some months and less oysters other months, please try to estimate how much you ate over the 12 months.

In the past year, how often on average did you eat oysters that were caught in the Gulf?

Now I would like to ask you to estimate the amount of GULF oysters that other members of your household eat.

Let's start with the other adults in your household. Please think about how often ____ (adult 1) ate oysters FROM THE GULF during the past <u>year</u>. If he/she ate more oysters some months and less oysters other months, please try to estimate how much he/she ate over the 12 months.

In the past year, how often on average did he/she eat oysters that was caught in the Gulf?

Thank you. When he/she ate oysters from the Gulf, how much did he/she typically eat? Please use these pictures to help you best estimate the amount he/she usually ate.

(If there are additional adults, repeat the above questions about oysters.)

You indicated there are ___ children in your household. Please think about how often ____ (child 1) ate **Oysters** FROM THE GULF during the past <u>year</u>. If he/she ate more oysters some months and less oysters other months, please try to estimate how much he/she ate over the past 12 months.

In the past year, how often on average did he/she eat oysters that was caught in the Gulf?
Thank you. When he/she ate oysters from the Gulf, how much did he/she typically eat? Please use these pictures to help you best estimate the amount he/she usually ate.

GENERAL QUESTIONS

In general, how safe did you think seafood was *before* the Gulf of Mexico oil spill? (choose one)

☐ Not safe at all

☐ Somewhat safe

☐ Safe

☐ Very safe

☐ Completely safe

Do you think that the safety of seafood is different now, after the Gulf of Mexico oil spill, than before the oil spill? (choose one)

☐ Yes, it's far less safe now

☐ Yes, it's a little less safe now

☐ No, it's about the same

☐ Yes, it's a little safer now

☐ Yes, it's far safer now

Have you noticed the presence of any oil at this location?

A. before the oil spill? ☐ Yes. ☐ No
B. after the oil spill ☐ Yes. ☐ No
C. in the last month? ☐ Yes. ☐ No

Since the Deepwater Horizon oil spill, has there been a change in the type of Gulf seafood you consume?

☐**Yes**

☐ Eat less (species code______________)

☐ Eat more (species code______________)

☐ Stopped eating (species code______________)

☐ Starting eating (species code______________)

☐Other____

Reason for change?________________________

☐ **No**, continue

Do you, or your family, consider seafood caught from this location, as an important source of food?

☐ Not important at all

☐ Somewhat important

☐ Important

☐ Very important

☐ Essential

How would you describe the amount of seafood that you eat from the Gulf of Mexico now compared with the amount of seafood that you ate from the Gulf prior to the Deepwater Horizon oil spill?

☐ Much less now

☐ Somewhat less now

☐ About the same

☐ Somewhat more now

☐ Much more now

How much risk does the Deepwater Horizon oil spill pose to human health?

☐ No risk at all

☐ Minimal risk

☐ Undecided/unsure

☐ Some risk

☐ Very significant risk

How great of a risk do you think the Deepwater Horizon oil spill posed to the Gulf ecosystem?

☐ No risk at all

☐ Minimal risk

☐ Undecided/unsure

☐ Some risk

☐ Very significant risk

I and/or citizens from my community have control over any potential risks associated with Gulf seafood consumption.

- ☐ Strongly disagree
- ☐ Disagree somewhat
- ☐ Undecided/unsure
- ☐ Agree somewhat
- ☐ Strongly Agree

Eating seafood from the Gulf means exposing yourself to a risk you can control.

- ☐ Strongly disagree
- ☐ Disagree somewhat
- ☐ Undecided/unsure
- ☐ Agree somewhat
- ☐ Strongly Agree

How safe do you think seafood from the Gulf of Mexico is currently?

- ☐ Completely safe
- ☐ Somewhat safe
- ☐ Undecided/unsure
- ☐ Somewhat unsafe
- ☐ Not safe at all

#________________________

FISH

In a typical season when you eat FISH, how often and how much FISH from the Gulf do you consume?

		Participant	Person 2<br>________________	Person 3<br>________________	Person 4<br>________________	Person 5<br>________________	Person 6<br>________________	Person 7<br>________________
		Gender: M or F<br>Age: ________<br>Weight (lbs): ________<br>Preg/Nursing: Y or N	Gender: M or F<br>Age: ________<br>Weight (lbs): ________<br>Preg/Nursing: Y or N	Gender: M or F<br>Age: ________<br>Weight (lbs): ________<br>Preg/Nursing: Y or N	Gender: M or F<br>Age: ________<br>Weight (lbs): ________<br>Preg/Nursing: Y or N	Gender: M or F<br>Age: ________<br>Weight (lbs): ________<br>Preg/Nursing: Y or N	Gender: M or F<br>Age: ________<br>Weight (lbs): ________<br>Preg/Nursing: Y or N	Gender: M or F<br>Age: ________<br>Weight (lbs): ________<br>Preg/Nursing: Y or N
Frequency	Feb, Mar, April (Current season)	□ None<br>□ < once/month<br>___ Meals per month<br>___ Meals per week<br>___ Meals per day	□ None<br>□ < once/month<br>___ Meals per month<br>___ Meals per week<br>___ Meals per day	□ None<br>□ < once/month<br>___ Meals per month<br>___ Meals per week<br>___ Meals per day	□ None<br>□ < once/month<br>___ Meals per month<br>___ Meals per week<br>___ Meals per day	□ None<br>□ < once/month<br>___ Meals per month<br>___ Meals per week<br>___ Meals per day	□ None<br>□ < once/month<br>___ Meals per month<br>___ Meals per week<br>___ Meals per day	□ None<br>□ < once/month<br>___ Meals per month<br>___ Meals per week<br>___ Meals per day
	Nov, Dec, Jan (Last season)	□ None<br>□ < once/month<br>___ Meals per month<br>___ Meals per week<br>___ Meals per day	□ None<br>□ < once/month<br>___ Meals per month<br>___ Meals per week<br>___ Meals per day	□ None<br>□ < once/month<br>___ Meals per month<br>___ Meals per week<br>___ Meals per day	□ None<br>□ < once/month<br>___ Meals per month<br>___ Meals per week<br>___ Meals per day	□ None<br>□ < once/month<br>___ Meals per month<br>___ Meals per week<br>___ Meals per day	□ None<br>□ < once/month<br>___ Meals per month<br>___ Meals per week<br>___ Meals per day	□ None<br>□ < once/month<br>___ Meals per month<br>___ Meals per week<br>___ Meals per day
	YEAR	□ None<br>□ < once/month<br>___ Meals per month<br>___ Meals per week<br>___ Meals per day	□ None<br>□ < once/month<br>___ Meals per month<br>___ Meals per week<br>___ Meals per day	□ None<br>□ < once/month<br>___ Meals per month<br>___ Meals per week<br>___ Meals per day	□ None<br>□ < once/month<br>___ Meals per month<br>___ Meals per week<br>___ Meals per day	□ None<br>□ < once/month<br>___ Meals per month<br>___ Meals per week<br>___ Meals per day	□ None<br>□ < once/month<br>___ Meals per month<br>___ Meals per week<br>___ Meals per day	□ None<br>□ < once/month<br>___ Meals per month<br>___ Meals per week<br>___ Meals per day
Portion Size		□ **A** (1-3 ounces)<br>□ **B** (3-5 ounces)<br>□ **C** (5-7 ounces)<br>□ **D** (7-9 ounces)<br>□ **E** (9-11 ounces)<br>□ **F** (11-13 ounces)<br>□ **G** (13-15 ounces)<br>□ **>** 15 ounces _______	□ **A** (1-3 ounces)<br>□ **B** (3-5 ounces)<br>□ **C** (5-7 ounces)<br>□ **D** (7-9 ounces)<br>□ **E** (9-11 ounces)<br>□ **F** (11-13 ounces)<br>□ **G** (13-15 ounces)<br>□ **>** 15 ounces _______	□ **A** (1-3 ounces)<br>□ **B** (3-5 ounces)<br>□ **C** (5-7 ounces)<br>□ **D** (7-9 ounces)<br>□ **E** (9-11 ounces)<br>□ **F** (11-13 ounces)<br>□ **G** (13-15 ounces)<br>□ **>** 15 ounces _______	□ **A** (1-3 ounces)<br>□ **B** (3-5 ounces)<br>□ **C** (5-7 ounces)<br>□ **D** (7-9 ounces)<br>□ **E** (9-11 ounces)<br>□ **F** (11-13 ounces)<br>□ **G** (13-15 ounces)<br>□ **>** 15 ounces _______	□ **A** (1-3 ounces)<br>□ **B** (3-5 ounces)<br>□ **C** (5-7 ounces)<br>□ **D** (7-9 ounces)<br>□ **E** (9-11 ounces)<br>□ **F** (11-13 ounces)<br>□ **G** (13-15 ounces)<br>□ **>** 15 ounces _______	□ **A** (1-3 ounces)<br>□ **B** (3-5 ounces)<br>□ **C** (5-7 ounces)<br>□ **D** (7-9 ounces)<br>□ **E** (9-11 ounces)<br>□ **F** (11-13 ounces)<br>□ **G** (13-15 ounces)<br>□ **>** 15 ounces _______	□ **A** (1-3 ounces)<br>□ **B** (3-5 ounces)<br>□ **C** (5-7 ounces)<br>□ **D** (7-9 ounces)<br>□ **E** (9-11 ounces)<br>□ **F** (11-13 ounces)<br>□ **G** (13-15 ounces)<br>□ **>** 15 ounces _______

#______________________

SHRIMP

In a typical season when you eat SHRIMP, how often and how much SHRIMP from the Gulf do you consume?

		**Participant**	**Person 2**<br>________________	**Person 3**<br>________________	**Person 4**<br>________________	**Person 5**<br>________________	**Person 6**<br>________________	**Person 7**<br>________________
		Gender: M or F<br>Age: ________<br>Weight (lbs): ________<br>Preg/Nursing: Y or N	Gender: M or F<br>Age: ________<br>Weight (lbs): ________<br>Preg/Nursing: Y or N	Gender: M or F<br>Age: ________<br>Weight (lbs): ________<br>Preg/Nursing: Y or N	Gender: M or F<br>Age: ________<br>Weight (lbs): ________<br>Preg/Nursing: Y or N	Gender: M or F<br>Age: ________<br>Weight (lbs): ________<br>Preg/Nursing: Y or N	Gender: M or F<br>Age: ________<br>Weight (lbs): ________<br>Preg/Nursing: Y or N	Gender: M or F<br>Age: ________<br>Weight (lbs): ________<br>Preg/Nursing: Y or N
**Frequency**	**Feb, Mar, April** (current season)	☐ None<br>☐ < once/month<br>___ Meals per month<br>___ Meals per week<br>___ Meals per day	☐ None<br>☐ < once/month<br>___ Meals per month<br>___ Meals per week<br>___ Meals per day	☐ None<br>☐ < once/month<br>___ Meals per month<br>___ Meals per week<br>___ Meals per day	☐ None<br>☐ < once/month<br>___ Meals per month<br>___ Meals per week<br>___ Meals per day	☐ None<br>☐ < once/month<br>___ Meals per month<br>___ Meals per week<br>___ Meals per day	☐ None<br>☐ < once/month<br>___ Meals per month<br>___ Meals per week<br>___ Meals per day	☐ None<br>☐ < once/month<br>___ Meals per month<br>___ Meals per week<br>___ Meals per day
	**Nov, Dec, Jan** (last season)	☐ None<br>☐ < once/month<br>___ Meals per month<br>___ Meals per week<br>___ Meals per day	☐ None<br>☐ < once/month<br>___ Meals per month<br>___ Meals per week<br>___ Meals per day	☐ None<br>☐ < once/month<br>___ Meals per month<br>___ Meals per week<br>___ Meals per day	☐ None<br>☐ < once/month<br>___ Meals per month<br>___ Meals per week<br>___ Meals per day	☐ None<br>☐ < once/month<br>___ Meals per month<br>___ Meals per week<br>___ Meals per day	☐ None<br>☐ < once/month<br>___ Meals per month<br>___ Meals per week<br>___ Meals per day	☐ None<br>☐ < once/month<br>___ Meals per month<br>___ Meals per week<br>___ Meals per day
	**YEAR**	☐ None<br>☐ < once/month<br>___ Meals per month<br>___ Meals per week<br>___ Meals per day	☐ None<br>☐ < once/month<br>___ Meals per month<br>___ Meals per week<br>___ Meals per day	☐ None<br>☐ < once/month<br>___ Meals per month<br>___ Meals per week<br>___ Meals per day	☐ None<br>☐ < once/month<br>___ Meals per month<br>___ Meals per week<br>___ Meals per day	☐ None<br>☐ < once/month<br>___ Meals per month<br>___ Meals per week<br>___ Meals per day	☐ None<br>☐ < once/month<br>___ Meals per month<br>___ Meals per week<br>___ Meals per day	☐ None<br>☐ < once/month<br>___ Meals per month<br>___ Meals per week<br>___ Meals per day
**Portion Size**		☐ **A** (1-3 ounces)<br>☐ **B** (3-5 ounces)<br>☐ **C** (5-7 ounces)<br>☐ **D** (7-9 ounces)<br>☐ **E** (9-11 ounces)<br>☐ **F** (11-13 ounces)<br>☐ **G** (13-15 ounces)<br>☐ **H** (15-17 ounces)<br>☐ **>** 17 ounces _______	☐ **A** (1-3 ounces)<br>☐ **B** (3-5 ounces)<br>☐ **C** (5-7 ounces)<br>☐ **D** (7-9 ounces)<br>☐ **E** (9-11 ounces)<br>☐ **F** (11-13 ounces)<br>☐ **G** (13-15 ounces)<br>☐ **H** (15-17 ounces)<br>☐ **>** 17 ounces _______	☐ **A** (1-3 ounces)<br>☐ **B** (3-5 ounces)<br>☐ **C** (5-7 ounces)<br>☐ **D** (7-9 ounces)<br>☐ **E** (9-11 ounces)<br>☐ **F** (11-13 ounces)<br>☐ **G** (13-15 ounces)<br>☐ **H** (15-17 ounces)<br>☐ **>** 17 ounces _______	☐ **A** (1-3 ounces)<br>☐ **B** (3-5 ounces)<br>☐ **C** (5-7 ounces)<br>☐ **D** (7-9 ounces)<br>☐ **E** (9-11 ounces)<br>☐ **F** (11-13 ounces)<br>☐ **G** (13-15 ounces)<br>☐ **H** (15-17 ounces)<br>☐ **>** 17 ounces _______	☐ **A** (1-3 ounces)<br>☐ **B** (3-5 ounces)<br>☐ **C** (5-7 ounces)<br>☐ **D** (7-9 ounces)<br>☐ **E** (9-11 ounces)<br>☐ **F** (11-13 ounces)<br>☐ **G** (13-15 ounces)<br>☐ **H** (15-17 ounces)<br>☐ **>** 17 ounces _______	☐ **A** (1-3 ounces)<br>☐ **B** (3-5 ounces)<br>☐ **C** (5-7 ounces)<br>☐ **D** (7-9 ounces)<br>☐ **E** (9-11 ounces)<br>☐ **F** (11-13 ounces)<br>☐ **G** (13-15 ounces)<br>☐ **H** (15-17 ounces)<br>☐ **>** 17 ounces _______	☐ **A** (1-3 ounces)<br>☐ **B** (3-5 ounces)<br>☐ **C** (5-7 ounces)<br>☐ **D** (7-9 ounces)<br>☐ **E** (9-11 ounces)<br>☐ **F** (11-13 ounces)<br>☐ **G** (13-15 ounces)<br>☐ **H** (15-17 ounces)<br>☐ **>** 17 ounces _______

#______________________

OYSTER

In a typical season when you eat OYSTERS, how often and how many OYSTERS from the Gulf do you consume?

		Participant	Person 2<br>________________	Person 3<br>________________	Person 4<br>________________	Person 5<br>________________	Person 6<br>________________	Person 7<br>________________
		Gender: M or F<br>Age: ________<br>Weight (lbs): ________<br>Preg/Nursing: Y or N	Gender: M or F<br>Age: ________<br>Weight (lbs): ________<br>Preg/Nursing: Y or N	Gender: M or F<br>Age: ________<br>Weight (lbs): ________<br>Preg/Nursing: Y or N	Gender: M or F<br>Age: ________<br>Weight (lbs): ________<br>Preg/Nursing: Y or N	Gender: M or F<br>Age: ________<br>Weight (lbs): ________<br>Preg/Nursing: Y or N	Gender: M or F<br>Age: ________<br>Weight (lbs): ________<br>Preg/Nursing: Y or N	Gender: M or F<br>Age: ________<br>Weight (lbs): ________<br>Preg/Nursing: Y or N
**Frequency**	**Feb, Mar, April** (Current season)	☐ None<br>☐ < once/month<br>___ Meals per month<br>___ Meals per week<br>___ Meals per day	☐ None<br>☐ < once/month<br>___ Meals per month<br>___ Meals per week<br>___ Meals per day	☐ None<br>☐ < once/month<br>___ Meals per month<br>___ Meals per week<br>___ Meals per day	☐ None<br>☐ < once/month<br>___ Meals per month<br>___ Meals per week<br>___ Meals per day	☐ None<br>☐ < once/month<br>___ Meals per month<br>___ Meals per week<br>___ Meals per day	☐ None<br>☐ < once/month<br>___ Meals per month<br>___ Meals per week<br>___ Meals per day	☐ None<br>☐ < once/month<br>___ Meals per month<br>___ Meals per week<br>___ Meals per day
	**Nov, Dec, Jan** (Last season)	☐ None<br>☐ < once/month<br>___ Meals per month<br>___ Meals per week<br>___ Meals per day	☐ None<br>☐ < once/month<br>___ Meals per month<br>___ Meals per week<br>___ Meals per day	☐ None<br>☐ < once/month<br>___ Meals per month<br>___ Meals per week<br>___ Meals per day	☐ None<br>☐ < once/month<br>___ Meals per month<br>___ Meals per week<br>___ Meals per day	☐ None<br>☐ < once/month<br>___ Meals per month<br>___ Meals per week<br>___ Meals per day	☐ None<br>☐ < once/month<br>___ Meals per month<br>___ Meals per week<br>___ Meals per day	☐ None<br>☐ < once/month<br>___ Meals per month<br>___ Meals per week<br>___ Meals per day
	**YEAR**	☐ None<br>☐ < once/month<br>___ Meals per month<br>___ Meals per week<br>___ Meals per day	☐ None<br>☐ < once/month<br>___ Meals per month<br>___ Meals per week<br>___ Meals per day	☐ None<br>☐ < once/month<br>___ Meals per month<br>___ Meals per week<br>___ Meals per day	☐ None<br>☐ < once/month<br>___ Meals per month<br>___ Meals per week<br>___ Meals per day	☐ None<br>☐ < once/month<br>___ Meals per month<br>___ Meals per week<br>___ Meals per day	☐ None<br>☐ < once/month<br>___ Meals per month<br>___ Meals per week<br>___ Meals per day	☐ None<br>☐ < once/month<br>___ Meals per month<br>___ Meals per week<br>___ Meals per day
**Portion Size**	**NUMBER OF OYSTERS**	☐ 1-6 oysters<br>☐ 7-12 oysters (1 doz)<br>☐ 13-18 oysters<br>☐ 19-24 oysters (2 doz)<br>☐ 25-30 oysters<br>☐ 31-36 oysters (3 doz)<br>☐ 37-42 oysters<br>☐ 43-48 oysters (4 doz)<br>☐ > 48 oysters ________	☐ 1-6 oysters<br>☐ 7-12 oysters (1 doz)<br>☐ 13-18 oysters<br>☐ 19-24 oysters (2 doz)<br>☐ 25-30 oysters<br>☐ 31-36 oysters (3 doz)<br>☐ 37-42 oysters<br>☐ 43-48 oysters (4 doz)<br>☐ > 48 oysters ______	☐ 1-6 oysters<br>☐ 7-12 oysters (1 doz)<br>☐ 13-18 oysters<br>☐ 19-24 oysters (2 doz)<br>☐ 25-30 oysters<br>☐ 31-36 oysters (3 doz)<br>☐ 37-42 oysters<br>☐ 43-48 oysters (4 doz)<br>☐ > 48 oysters ______	☐ 1-6 oysters<br>☐ 7-12 oysters (1 doz)<br>☐ 13-18 oysters<br>☐ 19-24 oysters (2 doz)<br>☐ 25-30 oysters<br>☐ 31-36 oysters (3 doz)<br>☐ 37-42 oysters<br>☐ 43-48 oysters (4 doz)<br>☐ > 48 oysters ______	☐ 1-6 oysters<br>☐ 7-12 oysters (1 doz)<br>☐ 13-18 oysters<br>☐ 19-24 oysters (2 doz)<br>☐ 25-30 oysters<br>☐ 31-36 oysters (3 doz)<br>☐ 37-42 oysters<br>☐ 43-48 oysters (4 doz)<br>☐ > 48 oysters ______	☐ 1-6 oysters<br>☐ 7-12 oysters (1 doz)<br>☐ 13-18 oysters<br>☐ 19-24 oysters (2 doz)<br>☐ 25-30 oysters<br>☐ 31-36 oysters (3 doz)<br>☐ 37-42 oysters<br>☐ 43-48 oysters (4 doz)<br>☐ > 48 oysters ______	☐ 1-6 oysters<br>☐ 7-12 oysters (1 doz)<br>☐ 13-18 oysters<br>☐ 19-24 oysters (2 doz)<br>☐ 25-30 oysters<br>☐ 31-36 oysters (3 doz)<br>☐ 37-42 oysters<br>☐ 43-48 oysters (4 doz)<br>☐ > 48 oysters ______

#________________________

BLUE CRAB

In the last 7 days, or in a typical week when you eat BLUE CRABS, how often and how many CRABS from the Gulf do you consume?

		Participant	Person 2	Person 3	Person 4	Person 5	Person 6	Person 7
			________________	________________	________________	________________	________________	________________
		Gender: M or F<br>Age: ________<br>Weight (lbs): ________<br>Preg/Nursing: Y or N	Gender: M or F<br>Age: ________<br>Weight (lbs): ________<br>Preg/Nursing: Y or N	Gender: M or F<br>Age: ________<br>Weight (lbs): ________<br>Preg/Nursing: Y or N	Gender: M or F<br>Age: ________<br>Weight (lbs): ________<br>Preg/Nursing: Y or N	Gender: M or F<br>Age: ________<br>Weight (lbs): ________<br>Preg/Nursing: Y or N	Gender: M or F<br>Age: ________<br>Weight (lbs): ________<br>Preg/Nursing: Y or N	Gender: M or F<br>Age: ________<br>Weight (lbs): ________<br>Preg/Nursing: Y or N
**Frequency**	**Feb, Mar, April (Current season)**	☐ None<br>☐ < once/month<br>___ Meals per month<br>___ Meals per week<br>___ Meals per day	☐ None<br>☐ < once/month<br>___ Meals per month<br>___ Meals per week<br>___ Meals per day	☐ None<br>☐ < once/month<br>___ Meals per month<br>___ Meals per week<br>___ Meals per day	☐ None<br>☐ < once/month<br>___ Meals per month<br>___ Meals per week<br>___ Meals per day	☐ None<br>☐ < once/month<br>___ Meals per month<br>___ Meals per week<br>___ Meals per day	☐ None<br>☐ < once/month<br>___ Meals per month<br>___ Meals per week<br>___ Meals per day	☐ None<br>☐ < once/month<br>___ Meals per month<br>___ Meals per week<br>___ Meals per day
	**Nov, Dec, Jan (Last season)**	☐ None<br>☐ < once/month<br>___ Meals per month<br>___ Meals per week<br>___ Meals per day	☐ None<br>☐ < once/month<br>___ Meals per month<br>___ Meals per week<br>___ Meals per day	☐ None<br>☐ < once/month<br>___ Meals per month<br>___ Meals per week<br>___ Meals per day	☐ None<br>☐ < once/month<br>___ Meals per month<br>___ Meals per week<br>___ Meals per day	☐ None<br>☐ < once/month<br>___ Meals per month<br>___ Meals per week<br>___ Meals per day	☐ None<br>☐ < once/month<br>___ Meals per month<br>___ Meals per week<br>___ Meals per day	☐ None<br>☐ < once/month<br>___ Meals per month<br>___ Meals per week<br>___ Meals per day
	**YEAR**	☐ None<br>☐ < once/month<br>___ Meals per month<br>___ Meals per week<br>___ Meals per day	☐ None<br>☐ < once/month<br>___ Meals per month<br>___ Meals per week<br>___ Meals per day	☐ None<br>☐ < once/month<br>___ Meals per month<br>___ Meals per week<br>___ Meals per day	☐ None<br>☐ < once/month<br>___ Meals per month<br>___ Meals per week<br>___ Meals per day	☐ None<br>☐ < once/month<br>___ Meals per month<br>___ Meals per week<br>___ Meals per day	☐ None<br>☐ < once/month<br>___ Meals per month<br>___ Meals per week<br>___ Meals per day	☐ None<br>☐ < once/month<br>___ Meals per month<br>___ Meals per week<br>___ Meals per day
**Portion Size**	**NUMBER OF CRABS**	☐ 1-3 crabs ☐ A<br>☐ 4-6 crabs ☐ B<br>☐ 7-12 crabs ☐ C<br>☐ 10-12 crabs ☐ D<br>☐ 13-18 crabs ☐ E<br>☐ 19-24 crabs ☐ F<br>☐ 25-30 crabs<br>☐ 31-36 crabs<br>☐ > 36 crabs ________	☐ 1-3 crabs ☐ A<br>☐ 4-6 crabs ☐ B<br>☐ 7-12 crabs ☐ C<br>☐ 10-12 crabs ☐ D<br>☐ 13-18 crabs ☐ E<br>☐ 19-24 crabs ☐ F<br>☐ 25-30 crabs<br>☐ 31-36 crabs<br>☐ > 36 crabs ________	☐ 1-3 crabs ☐ A<br>☐ 4-6 crabs ☐ B<br>☐ 7-12 crabs ☐ C<br>☐ 10-12 crabs ☐ D<br>☐ 13-18 crabs ☐ E<br>☐ 19-24 crabs ☐ F<br>☐ 25-30 crabs<br>☐ 31-36 crabs<br>☐ > 36 crabs ________	☐ 1-3 crabs ☐ A<br>☐ 4-6 crabs ☐ B<br>☐ 7-12 crabs ☐ C<br>☐ 10-12 crabs ☐ D<br>☐ 13-18 crabs ☐ E<br>☐ 19-24 crabs ☐ F<br>☐ 25-30 crabs<br>☐ 31-36 crabs<br>☐ > 36 crabs ________	☐ 1-3 crabs ☐ A<br>☐ 4-6 crabs ☐ B<br>☐ 7-12 crabs ☐ C<br>☐ 10-12 crabs ☐ D<br>☐ 13-18 crabs ☐ E<br>☐ 19-24 crabs ☐ F<br>☐ 25-30 crabs<br>☐ 31-36 crabs<br>☐ > 36 crabs ________	☐ 1-3 crabs ☐ A<br>☐ 4-6 crabs ☐ B<br>☐ 7-12 crabs ☐ C<br>☐ 10-12 crabs ☐ D<br>☐ 13-18 crabs ☐ E<br>☐ 19-24 crabs ☐ F<br>☐ 25-30 crabs<br>☐ 31-36 crabs<br>☐ > 36 crabs ________	☐ 1-3 crabs ☐ A<br>☐ 4-6 crabs ☐ B<br>☐ 7-12 crabs ☐ C<br>☐ 10-12 crabs ☐ D<br>☐ 13-18 crabs ☐ E<br>☐ 19-24 crabs ☐ F<br>☐ 25-30 crabs<br>☐ 31-36 crabs<br>☐ > 36 crabs ________
